# Clinical Management of Gingival Recessions with or Without Cervical Lesions: A Decisional Scheme Proposal

**DOI:** 10.3390/jcm14176134

**Published:** 2025-08-29

**Authors:** Luca Coccoluto, Stefano Speroni, Roberto Rotundo

**Affiliations:** Vita-Salute San Raffaele University, IRCCS San Raffaele, 20132 Milan, Italysperoni.stefano@hsr.it (S.S.)

**Keywords:** gingival recession, tooth abrasion, coronally advanced flap, connective tissue graft, tooth restoration

## Abstract

**Background**: Treatment of gingival recessions starts from an accurate diagnosis considering both periodontal tissue status and adjacent exposed dental tissues. Based on current scientific evidence and the authors’ clinical experience, a decisional scheme has been proposed for the management of gingival recession defects, with or without non-carious cervical lesions, taking into account gingival thickness and interproximal attachment levels. To illustrate its practical application, a series of representative clinical cases is presented, documenting the rationale and outcomes of the therapeutic decisions. **Methods**: According to the 2017 World Workshop Classification of Periodontal and Peri-Implant Diseases and Conditions, the gingival recession defect classifications have been used to build up a decision-making therapeutic process. Combined periodontal and restorative treatments in presence or absence of dental lesions have been performed. **Results**: In case of an identifiable cemento-enamel junction (CEJ) with or without non-carious cervical lesions (class A+ and class A−, respectively) and absence of interproximal attachment loss (RT1), flap approaches alone or in combination with connective tissue graft (CTG) were suggested. In case of an unidentifiable CEJ without cervical lesion (class B−), flap approaches alone were proposed in presence of adequate residual keratinized tissue (KT) and absence of interproximal attachment loss (RT1); if KT is extremely reduced, flap approaches + CTG may be performed. If the unidentifiable CEJ is associated with cervical lesions involving both root and crown surfaces (class B+), the combined restorative–periodontal treatment results as the most indicated approach. The adjunctive use of CTG should be also considered in presence of interproximal attachment loss (RT2 and RT3) and reduced gingival thickness (<1 mm). **Conclusions**: The proposed decisional scheme could be useful to address the clinicians during the decision-making process in the treatment of gingival recessions.

## 1. Introduction

The successful treatment of gingival recessions is represented by the complete root coverage (CRC) with the gingival margin coronal to cemento-enamel junction, combined with an optimal aesthetic outcome and a reduction or elimination of patient-perceived dentinal hypersensitivity [1,2]. The presence of gingival recessions, as conditions frequently requiring surgical treatment for aesthetic reasons, has been largely discussed [1,3,4,5]; however, few studies consider their subjective aesthetic impact [6,7]. Complete root coverage is considered the main clinical outcome for patients, general dentists, or periodontists [7]. On the other hand, the CRC alone is not sufficient to declare a full aesthetic success of the surgical procedures. In fact, other factors, such as the scalloped profile of gingival margin, absence of scars, perfect colour integration with the adjacent tissues, and the aligned mucogingival line must be taken into consideration to define a global concept of “success” in the treatment of gingival recession [8]. However, clinical success can only be achieved following a precise diagnostic assessment that integrates an aesthetic quantification of the smile with a periodontal analysis of the specific characteristics of the recession. In this context, an objective evaluation of the smile before and after treatment is essential to determine an aesthetically satisfactory outcome. Accordingly, the Smile Esthetic Index (SEI) represents a valid and reproducible method for assessing the aesthetic component of the smile [5].

In this context, owing to its diagnostic relevance, the 2017 World Workshop classification provides valuable information to support the clinical management and treatment planning of gingival recession deformities [9]. This treatment-oriented classification integrates both a diagnostic approach, used to assess gingival phenotype, recession defects, and associated cervical lesions, and a prognostic approach, which classifies gingival recession based on the extent of interdental clinical attachment loss.

The primary advantage of the current classification lies in its capacity to incorporate all the fundamental factors that assist the clinician in formulating an appropriate therapeutic strategy. On the other hand, gingival recession, defined as “the apical location of the gingival margin respect to the cemento-enamel junction”, is characterised by the damage of soft tissues associated with the exposition of the root along with attachment loss and bone [10].

The evaluation of the dental surface condition is therefore of paramount importance, as the loss of the cemento-enamel junction (CEJ) hinders accurate diagnosis, impairs prognostic assessment, and prevents reliable evaluation of treatment outcomes [11]. In such cases, the natural CEJ profile can be estimated using adjacent teeth, when not affected by cervical lesions, or the contralateral homologous tooth as a reference. This anatomical approximation has been recognised in the literature as a valid strategy to guide both restorative margin placement and the evaluation of gingival margin position during mucogingival surgical planning [12,13].

Frequently, root caries and non-carious cervical lesions are associated with gingival recession defects [14]. Bartlett and Shah classified the non-carious cervical lesions from the etiological point of view [15]. *Erosion* is defined as the loss of a broader, dish-shaped, and shallower lesion due to chemical action not involving bacteria. *Abrasion* is the loss of tooth substance characterised by a different degree of sharply defined margins caused by traumatic toothbrushing and abrasive toothpaste. *Abfraction* is a wedge-shaped cervical lesion that cannot be explained by toothbrush abrasion or erosion alone. These non-carious cervical lesions may involve the root cementum and/or the enamel with a partial or total loss of the cemento-enamel junction.

Furthermore, the condition of the dental surface defects may cause some technical difficulties during the surgical procedures, i.e., the positioning and the stability of the flap/graft on the exposed root. Thus, predictable complete root coverage might not be obtained even in gingival recession defects associated with deep root and crown defects. Nevertheless, surgical approaches have been proposed to treat single or multiple gingival recessions associated with non-carious cervical lesions.

Some techniques have demonstrated the potential to achieve successful clinical outcomes using only a coronally advanced flap (CAF) or by combining CAF with a single or double layer of connective tissue graft (CTG) [16]. According to a systematic review and network meta-analysis by Chambrone et al. [17], subepithelial connective tissue graft (SCTG) plus a coronally advanced flap (CAF) can be considered the gold standard for the treatment of single gingival recession defects.

More recently, combined restorative and periodontal approaches have shown more favourable results in treating gingival recession associated with non-carious cervical lesions (NCCLs) [18,19]. Similarly, tunnelling approaches have recently gained significant popularity among clinicians due to their promising clinical and aesthetic outcomes in the treatment of gingival recession defects, in particular for multiple gingival recessions [20]. Further modifications, such as the laterally closed tunnel (LCT), have been successfully employed in the management of isolated, deep mandibular recessions, leading to predictable coverage (mean root coverage of >90%) of RT1 defects [21].

Therefore, a global diagnosis of the entire gingival recession area—encompassing both periodontal and dental tissue components—is essential and must be integrated into the treatment planning of these defects. However, within such a heterogeneous therapeutic landscape, greater clarity is warranted. To this end, a decision-making scheme is proposed through the presentation of selected clinical cases, with the aim of guiding the management of gingival recession defects, both with and without non-carious cervical lesions.

## 2. Methods: Decisional Tree Proposal

A logical and evidence-based clinical rationale is presented, grounded in a comprehensive diagnostic assessment of the recession area and aligned with the 2017 World Workshop Classification of Periodontal and Peri-Implant Diseases and Conditions concerning mucogingival deformities [9]. Central to this approach is the integration of the periodontal evaluation of the gingival recession with the structural analysis of the dental hard tissues. This combined assessment supports the development of a decision-making scheme aimed at guiding clinicians in selecting the most appropriate treatment following an accurate diagnosis (Table 1).

### 2.1. Periodontal Evaluation

The 2017 World Workshop Classification emphasises the evaluation of gingival phenotype, recession severity, and the presence of associated cervical lesions [9]. The first step involves the definition of gingival phenotypes, considering that this aspect can vary considerably both among individuals and within different sites in the same individual. Notably, thin phenotypes (defined as <1 mm) are associated with less predictable outcomes following root coverage procedures [13]. Similarly, beyond phenotype, recession depth and gingival thickness are critical determinants of treatment success. Increased recession depth is inversely correlated with the probability of achieving complete root coverage (CRC) following surgical intervention. Furthermore, the integrity of the interdental attachment and the width of keratinized tissue (KTW) are essential parameters influencing the final outcome. Based on clinical measurements of the interdental clinical attachment level, gingival recessions can be classified into three distinct types, which further guide treatment selection [23].

The evaluation of the surrounding keratinized tissues allows for planning the design of the flap, which should incorporate an adequate amount (>2 mm) of gingiva, such as a coronally advanced flap, laterally positioned flap, or bipedicle flaps. In the presence of a reduced amount of KT, the use of a soft tissue graft to increase or compensate the residual keratinized tissue should be considered.

### 2.2. Hard Dental Tissue Evaluation (Crown and Root Assessment)

Along with the periodontal evaluation that allows for appropriate planning of the surgical technique (design and type of the flap), the scheme suggests the assessment of the dental tissue condition involved in the recession area.

Pini Prato et al. [24] proposed a classification system based on the presence (A) or absence (B) of an identifiable CEJ and the presence (+) or absence (−) of non-carious cervical lesions.

In the presence of deep root lesions, the use of a bilaminar technique is recommended, as it enhances vascular support and flapstability. The bilaminar technique involves the placement of a connective tissue graft between the root surface and a coronally advanced flap, ensuring blood supply from both the underlying periosteum and the overlying flap. This dual-layered approach improves root coverage outcomes, tissue integration, and long-term aesthetic results [25,26,27].

In fact, a connective tissue graft was needed to fill in the abrasion, and the flap/graft can be easily stabilised. However, an appropriate prior assessment of the flap thickness (periodontal phenotype) is mandatory in order to make the most suitable choice.

If the abrasion is smooth and in presence of a thick keratinized tissue, a flap procedure (coronally advanced or laterally positioned flaps, or bipedicle flaps) is suggested. However, if the abrasion also involves the enamel, the gingival margin cannot be placed in correspondence with the coronal finishing line of the abrasion. In fact, if a complete root coverage occurs, a flat profile of the gingival margin will result in an unpleasant aesthetic outcome. In this case, it is mandatory to restore the lost enamel and to rebuild the CEJ by means of restorative materials [13,28]. Following the restoration of the crown, the exposed root may be covered by means of coronally advanced flap or bilaminar technique positioning the gingival margin slightly coronal to the re-built CEJ.

## 3. Results

### Case Reports

The following case series illustrates how a thorough diagnostic approach—encompassing both periodontal and dental hard tissue evaluation—can effectively guide the selection of the most appropriate therapeutic strategy. However, the lack of a control group, the subjective assessment of clinical outcomes, and the limited generalizability of individual cases represent inherent limitations of the present work, highlighting the need for future studies on larger patient cohorts to validate the proposed therapeutic decision-making scheme.

**Case # 1: Identifiable CEJ without non-carious cervical lesion (Class A−).**

**Figure 1:**
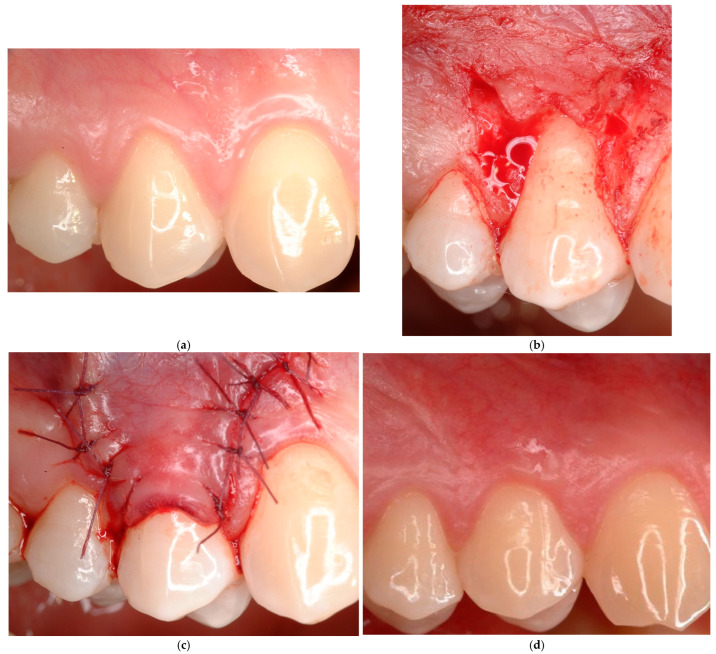
(**a**–**c**) Coronally advanced flap performed in class A− for dentinal hypersensitivity. (**d**) After 12-month follow-up, the gingival margin (GM) is located at the cement–enamel junction (CEJ) level.

In the presence of an identifiable CEJ without a surface discrepancy (Class A−), a coronally advanced flap (CAF) [29] may be considered as an appropriate approach for the treatment of this condition (Figure 1). This technique is particularly indicated in RT1 cases due to the presence of healthy interdental clinical attachment and a sufficient amount (at least 2 mm of width and 1 mm of thickness) of keratinized tissue apically to the recession. In the literature, systematic reviews [11] have demonstrated the efficacy and the predictability of this technique. In case of lack or reduced amount of keratinized tissue apical to the gingival recession, alternative approaches may be represented by the two-step procedure [30], the laterally positioned flap [31,32,33], the Double Papilla Flap [34], or a combined laterally and coronally advanced flap [35]. In case of RT2 gingival recession, the use of a bilaminar technique may be indicated [36,37,38,39].

**Case # 2: Identifiable CEJ with non-carious cervical lesion (Class A+).**

**Figure 2:**
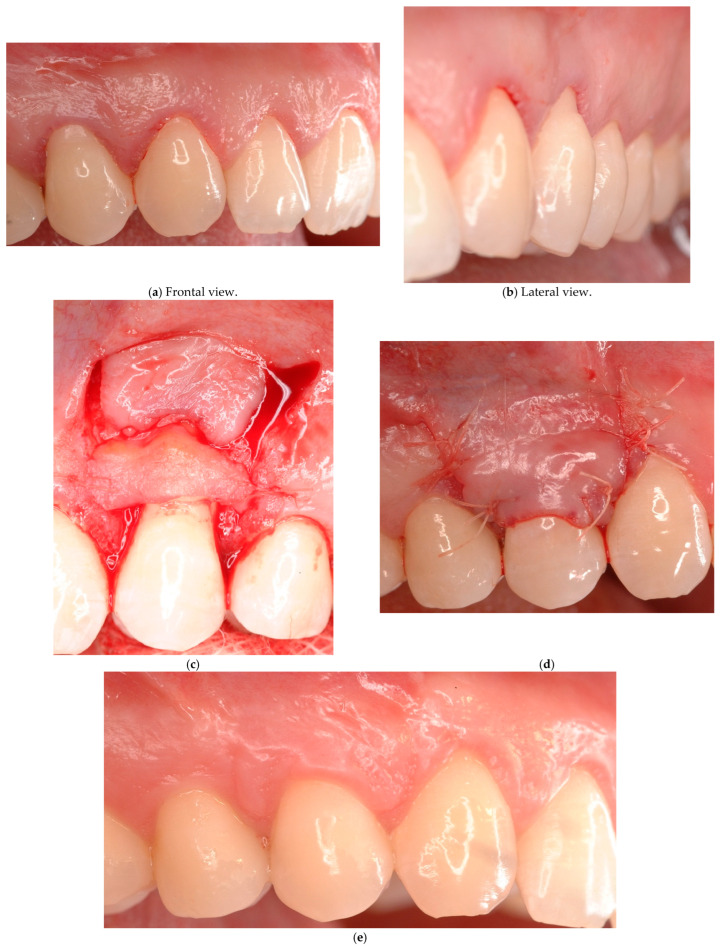
(**a**–**d**) Bilaminar technique performed in Class A+ for dentinal hypersensitivity associated with non-carious cervical lesions (NCCLs) at the root level. (**e**) After a 6-month follow-up, gingival margin (GM) is located at the cement–enamel junction (CEJ) level.

In the presence of an identifiable CEJ associated with a surface discrepancy that involves only the root surface (Class A+) and in the presence of RT1 and 2 gingival recession, the coronally advanced flap with connective tissue graft (bilaminar technique) [36,37,38,39] represents a reasonable approach (Figure 2). The connective tissue graft is aimed to fill in the root cementum abrasion, eliminating the discrepancy between the flap and the root surface thus increasing the stability of the tissues. In this case, the use of a connective tissue graft may also avoid the grinding of the sharp edges of the abrasion. In fact, further elimination of dental tissues may lead to the onset/increase in dental hypersensitivity if a complete root coverage is not achieved. The good adaptability of the graft onto the surface defect prevents the creation of dead tract underneath, too. In case of a very deep discrepancy, a double layer of connective tissue graft may be used as suggested by Pini Prato et al. [40].

**Case # 3: Unidentifiable CEJ without non-carious cervical lesion (Class B−).**

**Figure 3:**
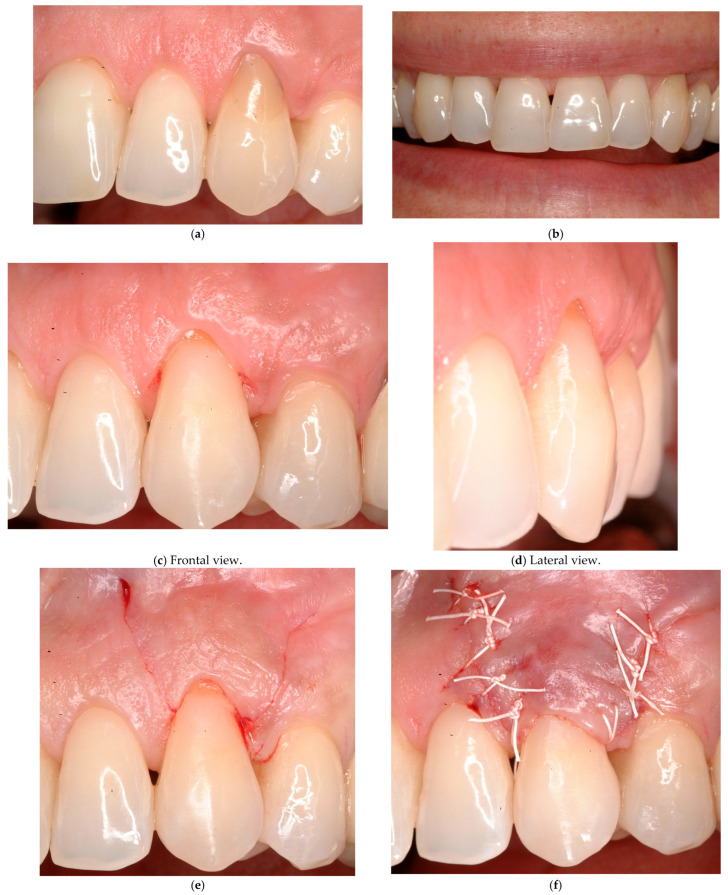

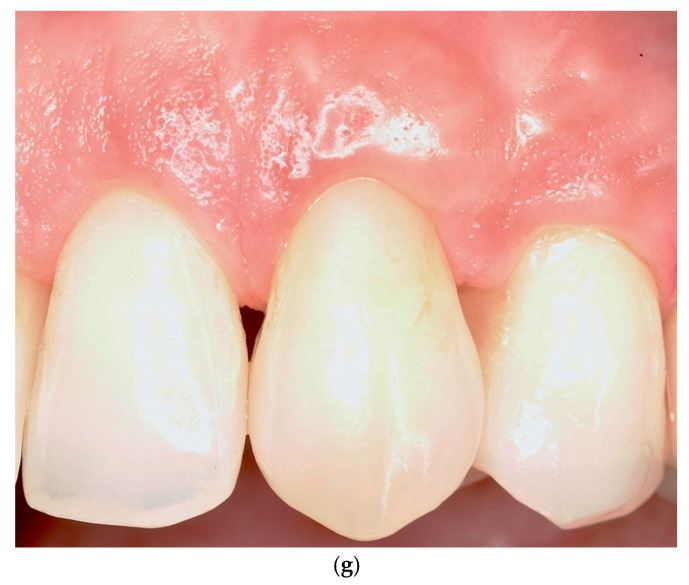
(**a**) Baseline: SEI score = 6. (**b**) Six-month control: SEI score = 7. (**c**–**f**) Coronally advanced flap performed in class B− for aesthetic concerns. (**g**) After a 6-month follow-up, the gingival margin (GM) is supposed to be located at the cement–enamel junction (CEJ) level. Therefore, it is not possible to establish if a complete root coverage is achieved.

In the presence of an unidentifiable CEJ due to a smooth abrasion without detectable surface discrepancy (Class B−) and an adequate gingival amount/thickness, the coronally advanced flap technique may be considered as an appropriate treatment choice (Figure 3) associated with a previous CEJ reconstruction with composite resin materials. A bilaminar technique may be used in the presence of a reduced amount of keratinized tissue width. In this situation, the absence of a specific referral point (CEJ) prevents the possibility of measuring the baseline recession depth and evaluating the final outcome. Therefore, the success of the treatment cannot be evaluated adequately. A surrogate referral point may be established mimicking the CEJ profile of the adjacent teeth and/or the one of the contra-lateral homologous teeth.

In these cases, the surgical intervention is aimed to reduce the recession depth avoiding the anaesthetic appearance of a “long tooth” and the possible related dental hypersensitivity.

If the amount of keratinized tissue width apically to the recession is not adequate, a laterally positioned or a bipapillary flap may be indicated with or without a connective tissue graft.

**Case # 4: Unidentifiable CEJ with non-carious cervical lesion (B+).**

**Figure 4:**
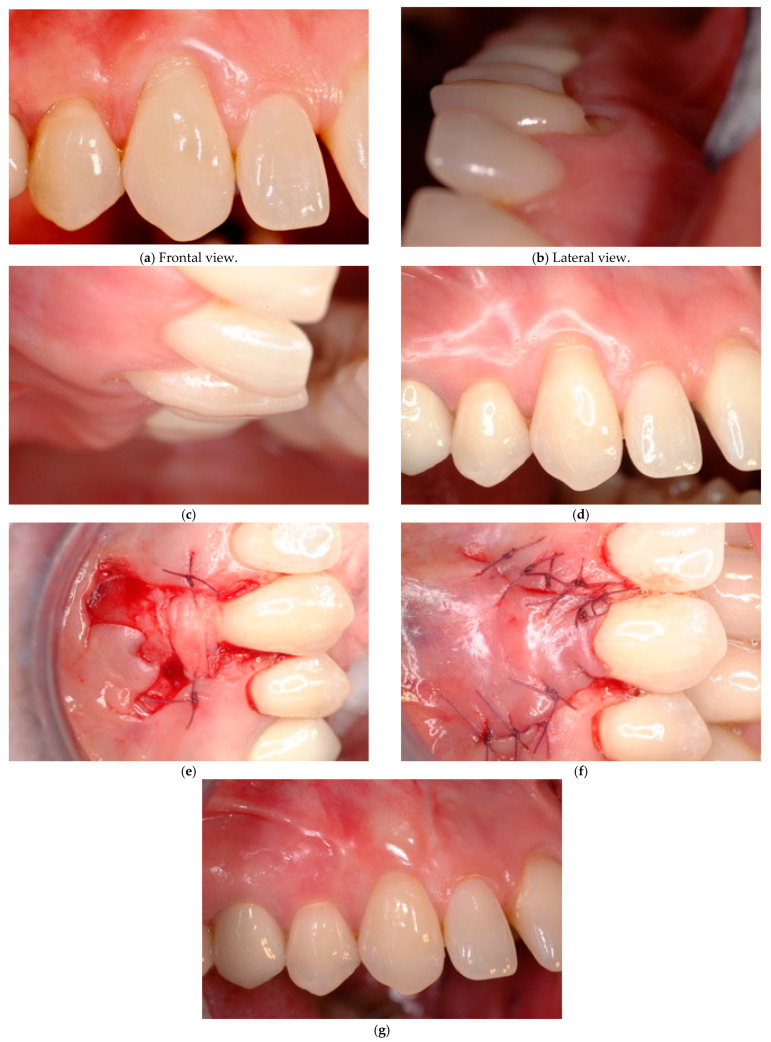
(**a**–**g**) Bilaminar technique plus enamel restoration performed in Class B+ for dentinal hypersensitivity and esthetic reason. Enamel restoration and CEJ reconstruction are performed before the surgical procedure. (**e**) Once the CEJ is restored, the gingival margin of the flap is positioned in correspondence with the new referral point (CEJ r). (**g**) After a 12-month follow-up, GM is located at the restored cement–enamel junction (CEJ r) level.

In the presence of an unidentifiable CEJ associated with a severe dental surface discrepancy involving the root and the crown (Class B+), a combined restorative–periodontal technique appears the most favourable approach to re-establish the physiological anatomy of the dental and the periodontal tissues, thus achieving a good aesthetic result (Figure 4).

The combined technique consists of an initial crown reconstruction by means of adhesive nanohybrid composite resin (selected for its superior polishability [41,42]), in order to restore the lost enamel and the related CEJ profile. Once the enamel/CEJ reconstruction has been performed, the morphology of the defect changes from Class B+ to Class A+. Consequently, if the residual root defect is shallow (<0.5 mm), a coronally advanced flap may be used, while, in the presence of a deep residual root discrepancy (≥0.5 mm), a coronally advanced flap with a connective tissue graft appears the most adequate approach [43]. In case of a very deep abrasion, the dental surface discrepancy could be filled in using a double layer of connective tissue grafts. This approach is quite different from the one proposed by other authors who suggested a reconstruction of the entire non-carious cervical lesion involving both the enamel and the root cementum followed by a coronally positioned flap [18,19].

## 4. Discussion

Several surgical approaches have been proposed in the literature to treat gingival recession defects. The selection of one rather than another surgical technique depends on several factors related to the affected area, such as the size of the recession defect, the presence or absence of keratinized tissue adjacent to the defect, the width and height of the interdental soft tissue, the root surface condition, the tooth position, the depth of the vestibulum, or the presence of frenula [13,44]. These anatomical conditions have a direct influence on the prognosis and stability of the surgical outcome and should therefore be carefully evaluated during treatment planning. Among patient-related factors, particular consideration should be given to minimising the number of surgeries and intraoral surgical sites, as well as meeting the patient’s aesthetic expectations [45]. In this view, when single or multiple recessions affect teeth, they should be treated at the same surgical time. Coronally advanced flap and tunnelling procedures combined with a connective tissue graft are considered the most predictable treatment options for single and multiple gingival recession defects (RT I and RT II) [9,11,25,46,47,48]. However, the probability of achieving complete root coverage is not solely dependent on the surgical technique, but it is also closely associated with the intrinsic features of the recession defect (RT I and RT II). In fact, systematic reviews [11] report a wide range of variability in terms of percentage of root coverage both between and within the studies, indicating that the procedures are operator sensitive and/or that some factors (prognostic factors) influencing the treatment outcomes have not been investigated adequately during the diagnostic and surgical phases.

Recession defects are often caused by tooth-brushing trauma and require a root coverage procedure aimed to provide good aesthetic outcomes. These lesions are often associated with a great variability of root and/or crown abrasion, which may damage the CEJ. An unidentifiable CEJ causes difficulties during the diagnostic and therapeutic phases. In fact, if the CEJ is not identifiable, the main referral point is lost, and it is impossible to evaluate the depth and the width of the recession (diagnosis) and the final outcome (prognosis). In addition, the presence of a pronounced severe abrasion that affects the exposed root and the crown may impair the stability of the graft and the position of the overlaid flap during the suturing phase.

Therefore, a predictable complete root coverage might not be obtained and evaluated even in RT I and II defects associated with root and crown abrasion.

To address these challenges, a treatment-oriented scheme is proposed, designed to guide the selection of the most appropriate surgical approach based on a thorough and systematic diagnostic process. In fact, the aim of this article is to propose a clinical scheme to treat recession type I and II defects, taking into consideration both the conditions of dental and periodontal tissue through the presentation of the same cases. This scheme is presented in Table 1. Before initiating treatment for gingival recession defects, it is essential to identify the underlying etiological factors. Although the exact cause of gingival recession remains unclear [14], current evidence suggests a multifactorial origin. Therefore, the first step in an effective prevention and management strategy involves recognising both individual susceptibility and modifiable risk factors. Primary susceptibility factors include a thin gingival phenotype, a reduced or absent band of keratinized tissue (<2 mm), probing depths extending beyond the mucogingival junction, and a positive history of progressive recession and/or periodontal disease. Modifiable conditions contributing to gingival recession include plaque accumulation, chronic periodontal inflammation, aberrant frenulum insertion, traumatic oral hygiene habits, subgingival restoration margins, tobacco use, and systemic factors such as diabetes [49,50].

Non-surgical management should focus on eliminating or controlling these contributing factors. This involves the establishment of meticulous plaque control, correction or removal of subgingival overhanging restorations, behavioural modifications to reduce traumatic habits, and the use of desensitising agents where indicated. Following causal therapy, in order to plan a surgical approach, the first diagnostic step consists of an accurate periodontal evaluation using the 2017 World Workshop classification [9] and the assessment of the width/thickness of the adjacent gingiva (apical, mesial, and distal) in the recession area. This evaluation is needed to perform a proper flap design. The use of a coronally advanced flap (CAF) without a connective tissue graft (CTG) is generally not recommended in cases where interdental clinical attachment loss is present (RT2) or when there is a minimal width of keratinized tissue (<2 mm) apical to the gingival recession. Additional anatomical limitations, such as a high frenal insertion at the mucogingival junction or a shallow vestibular depth, may further compromise the outcome of CAF alone. In situations where the root is buccally displaced or when it shows a deep cervical defect, the inclusion of a CTG in conjunction with CAF is strongly advised to enhance soft tissue volume and improve the chances of root coverage. Sometimes, a preliminary adjunctive orthodontic treatment able to replace the root correctly within the bone envelope should be considered.

The CTG can also be successfully combined with tunnel techniques, achieving favourable outcomes in terms of complete root coverage (CRC) and mean root coverage (MRC) [21,51,52,53,54]. Recent comparative studies evaluating tunnel techniques combined with CTG versus CAF + CTG have demonstrated no statistically significant differences in clinical outcomes. Notably, a systematic review and meta-analysis by Tavelli et al. [20] reported that the tunnel technique, when compared to CAF, yielded superior results, particularly in maxillary sites and in RT I and RT II gingival recession defects. Similarly, another recent meta-analysis confirmed that the tunnel technique achieves clinical outcomes comparable to CAF in the management of multiple gingival recessions.

The clinical success of the tunnel technique is likely attributed to its use of a split-thickness flap and microsurgical refinement, which preserve vascular supply and minimise tissue trauma. The modified tunnel technique (mTUN) has been shown to effectively improve gingival recessions, and the presence of shallow non-carious cervical lesions (≤1 mm) does not appear to adversely affect surgical outcomes [55]. Overall, the inclusion of a CTG is associated with greater long-term gingival stability and more predictable root coverage compared to CAF alone [14].

The second diagnostic step takes into consideration the exposed root evaluating the presence of an identifiable or unidentifiable CEJ and the presence of a non-carious cervical lesion that may involve only the root or both root and enamel. This assessment allows for choosing the most appropriate periodontal surgical technique with or without enamel restoration.

Santamaria et al. demonstrated that gingival recession defects associated with non-carious cervical lesions (NCCLs) exhibited comparable clinical outcomes when treated with either a coronally advanced flap (CAF) alone or a CAF in combination with a connective tissue graft (CAF + CTG), regardless of whether the NCCLs were restored prior to surgery [18,19]. However, improved outcomes in terms of dentinal hypersensitivity reduction, gingival margin contour, and restoration longevity have been reported when a combined surgical and restorative approach is employed for the treatment of recession defects associated with NCCLs [18,43,56,57]. Based on the collective evidence and expert opinion, a combined approach appears to offer the most comprehensive and predictable results in such clinical scenarios [58,59].

Supporting this view, a prospective study by De Sanctis et al. [60] found no statistically significant differences in complete root coverage (CRC) outcomes between teeth with reconstructed cemento-enamel junctions (CEJs) and those with natural anatomical CEJs, further validating the clinical reliability of the restorative–surgical strategy. However, current evidence remains limited for several clinical scenarios, such as recessions on teeth with cervical lesions extending apically to the gingival margin, recessions associated with advanced interproximal attachment loss (RTIII), and multiple adjacent recessions with cervical defects in posterior regions. These areas warrant further prospective studies to validate and refine restorative–surgical treatment protocols [61].

Finally, the proposed clinical scheme could be useful to address the clinicians during the decision-making process in the treatment of gingival recession defects, considering all factors deemed to be strongly correlated with the treatment outcomes. In some circumstances, further clinical trials are needed to corroborate the suggested rationale.

## 5. Conclusions

The presence or absence of non-carious lesions in correspondence with the gingival recession area, the reduced amount of KT and gingival thickness, and the presence or absence of interproximal attachment loss call for a correct diagnosis to establish the most appropriate treatment approach. Therefore, a decisional scheme that takes into consideration several surgical and non-surgical approaches has been proposed in order to facilitate clinicians’ decisional process for treating gingival recessions with or without cervical lesions based on the current literature.

## Figures and Tables

**Table 1:** A treatment-oriented scheme, based on a thorough and systematic diagnostic process.

Gingival Recession Defects			Periodontal Tissue Evaluation					
			KT		GT		IA	
			>2 mm	<2 mm	>1 mm	<1 mm	−	+
**Dental surface defect evaluation**	Class A (CEJ identifiable)	+ (discrepancy)	Flaps + CTG	Flaps + CTG	Flaps + (depth discrepancy assessment)	Flaps + CTG	Flaps + CTG	Flaps + CTG
		− (No discrepancy)	Flaps	Flaps + CTG	Flaps	Flaps + CTG	Flaps	Flaps + CTG
	Class B (CEJ unidentifiable)	+ (discrepancy)	Flaps + CTG + Enamel restoration	Flaps + CTG + Enamel restoration	Flaps + CTG + Enamel restoration	Flaps + CTG + Enamel restoration	Flaps + CTG + Enamel restoration	Flaps + CTG + Enamel restoration
		− (No discrepancy)	Flaps	Flaps + CTG	Flaps	Flaps + CTG	Flaps	Flaps + CTG

KT: keratinised tissue; GT: gingival thickness; IA: loss of interdental attachment (“−”: absence of loss of IA; “+”: presence of loss of IA); CEJ: Cemento-Enamel Junction; flaps: coronally advanced flap (CAF) or tunnelling approach (TUN); and CTG: connective tissue graft. The tunnelling technique can be considered as an alternative option in appropriate clinical indications. Discrepancy: Clinically detectable morphological alteration (step or concavity) on the root surface, evaluated by direct observation and probing (+ = Discrepancy depth > 0.5 mm). Gingival thickness should be assessed preoperatively using the probe transparency method, whereby visibility of a periodontal probe through the gingival margin indicates a thin phenotype, whereas its invisibility suggests a thick phenotype [22]. In the presence of a root surface discrepancy or a thin gingival phenotype (GT < 1 mm), the application of a connective tissue graft is essential to ensure long-term periodontal stability. For thin phenotypes, the depth of the discrepancy influences the required thickness of the graft. Conversely, in cases with a thick gingival phenotype, the indication for connective tissue grafting should be determined by the extent of the discrepancy. Generally, the greater the extent of the discrepancy (within the limits of the tooth’s prognosis), the stronger the recommendation for grafting, as it serves to prevent flap collapse into the defect.

## Data Availability

The original contributions presented in this study are included in the article. Further inquiries can be directed to the corresponding author.

## Abbreviations

The following abbreviations are used in this manuscript:

CAF	Coronally advanced flap
CEJ	Cemento-enamel junction
CRC	Complete root coverage
CTG	Connective tissue graft
IA	Interdental attachment
KT	Keratinized tissue
KTW	Width of keratinized tissue
LCT	Laterally closed tunnel
NCCL	Non-carious cervical lesion
RT	Recession type
TUN	Tunnel technique
